# First record of the family Ameronothridae (Acari: Oribatida) from Japan – new species, juvenile morphology, ecology and biogeographic remarks

**DOI:** 10.1080/01647954.2019.1629624

**Published:** 2019-06-23

**Authors:** Tobias Pfingstl, Shimpei F. Hiruta, Maximilian Wagner, Wataru Hagino, Satoshi Shimano

**Affiliations:** a Department for Biodiversity and Evolution, Institute of Biology, University of Graz, Graz, Austria; b Center for Molecular Biodiversity Research, National Museum of Nature and Science, Tsukuba, Japan; c Department of Bioresources Engineering, National Institute of Technology, Okinawa College, Nago-City, Japan; d Department of Intercultural Communication, Science Research Center, Hosei University, Tokyo, Japan

**Keywords:** Littoral, ontogeny, Hokkaido, systematics, cold temperate zone

## Abstract

The Ameronothridae are recorded for the first time from Japanese coasts with the new species *Ameronothrus yoichi* sp. n. from Hokkaido. The report of this species represents the most southern occurrence of an *Ameronothrus* species in the Asian Pacific region. *Ameronothrus yoichi* sp. n. can be easily distinguished from its congeners by the conspicuously pusticulate body surface and the loss of dorsal companion setae *d* on all genua in the adult stage. Based on adult and juvenile morphology, a close relation to *Ameronothrus maculatus* and *A. schneideri* is suggested. *Ameronothrus yoichi* sp. n. is classified as a lichenivorous inhabitant of sediment-free rocky coastal substrates. Due to a lack of genetic sequence data of nearly all ameronothrid species a molecular genetic comparison is yet unfeasible, but a Bayesian inference tree based on the 18S rRNA gene shows a paraphyletic clustering of the ameronothrid *A. yoichi* sp. n. and *Paraquanothrus grahami*.

http://www.zoobank.org/urn:lsid:zoobank.org:pub:5B772E2C-7D5E-4C86-9955-AB84A84C50DA

## Graphical abstract

**Figure:**
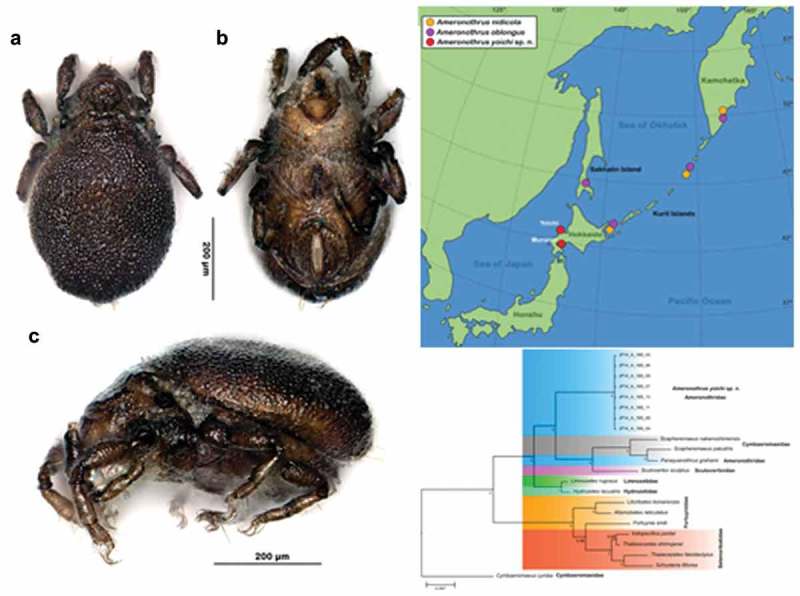

## Introduction

The Ameronothridae are a group of several genera of freshwater and marine associated oribatid mites that either live in ephemeral rock pools of terrestrial environments or in various habitats of coastal regions. The taxonomic history of this family was subject to many controversies and confusing changes so that differing concepts still can be found. Berlese early proposed the genus *Ameronothrus* Berlese, 1896 and included it in Carabodidae (Berlese 1896), but later Willmann (1931) noticed that this genus represents an independent taxon. Though the latter author is often given as family author in literature (e.g. Weigmann 2006), it was Vitzthum (1942) who first mentioned the name Ameronothridae and hence is the true author of this taxon (e.g. Pfingstl 2017). Decades later, Schulte (1975) noticed remarkable morphological similarities between the northern hemispheric marine associated Ameronothridae, till then only consisting of the single genus *Ameronothrus*, and the southern hemispheric marine associated Podacaridae, containing the genera *Podacarus, Halozetes, Alaskozetes, Antarcticola* and *Pseudantarcticola*, therefore he suggested merging the two families. Based on reasonable argumentation, Weigmann and Schulte (1977) supported his suggestion and hence included all the former Podacaridae but also the freshwater associated South African Aquanothridae in the family Ameronothridae and this concept was followed by most subsequent literatures (e.g. Subías 2004; Norton and Behan-Pelletier 2009). Nevertheless, Pfingstl (2017) questioned this taxonomic rearrangement highlighting molecular genetic and biogeographic studies that indicated an independent evolutionary origin of Ameronothridae and Podacaridae. Consequently, the latest concept of Ameronothridae (Norton and Franklin 2018) again excludes the Podacaridae, but retains the freshwater associated genera, *Aquanothrus, Paraquanothrus* and *Chudalupia* as subfamily Aquanothrinae besides the marine associated *Ameronothrus*. Due to this confusing background, we will mainly focus in this paper on the genus *Ameronothrus*, which represents the northern hemispheric littoral members of the Ameronothridae.

*Ameronothrus* presently contains 13 species (Subías 2004) that inhabit various coastal habitats, as, for example, rocky intertidal shores, salt marshes, limnic estuary zones, but also terrestrial habitats on further inlands (Schulte et al. 1975). They are known to feed on a wide variety of food, including lichen, algae and fungi (Schulte 1976). Some species are stenotopic, others are holeurytopic inhabitants of marine-associated environments, whereas species at lower latitudes show greater affinity for littoral habitats and species found at higher latitudes are mainly restricted to terrestrial habitats (Schulte et al. 1975; Marshall and Convey 2004).

Apart from single exceptional records in the Caribbean (Willmann 1936) and on the South African coast (Weigmann 1975), *Ameronothrus* species are distributed in the Holarctic (Schulte 1975), where they predominantly occur within cold-temperate regions (e.g. Procheş and Marshall 2001). Most records are from European coasts of the Northern and Baltic Sea, but there are also numerous reports from European Atlantic shores, as well as from polar coasts of Greenland and other arctic islands. Further comprehensive findings stretch along the Pacific North American coast from Alaska to California (Schulte 1975), whereas few records are known from the Atlantic North American coast and from the Mediterranean Sea (Schulte 1975; Pfingstl 2017). For a long time, the Asian Pacific shorelines seemed to be devoid of *Ameronothrus*, until two species *Ameronothrus nidicola* Sitnikova, 1975 and *Ameronothrus oblongus* Sitnikova, 1975 were discovered in the Far East of Russia, on Kamchatka (Sitnikova 1975), and later they were also reported from the Kuril Islands (e.g. Klimov 1998). However, although there is important subsequent literature on the systematics of Ameronothridae (Krause et al. 2016; Pfingstl 2017; Norton and Franklin 2018) and on further records from various Holarctic locations (e.g. Bücking et al. 1998; Søvik 2003), no further *Ameronothrus* species have been discovered and published for the last four decades.

In the course of an ongoing project investigating the intertidal mite fauna of Japanese coasts, we found *Ameronothrus* individuals on the shore of Hokkaido. Therefore, aim of the present paper is, first, to assess the taxonomic status of the found individuals; second, provide a detailed description; third, give information on juvenile morphology and fourth, discuss the biogeographic aspects of this record.

## Material and methods

Samples of littoral algae and lichens were scraped off rocks and concrete walls with a knife or a small shovel and then put in Berlese-Tullgren funnels for about 24 h to extract mites. Afterwards, collected specimens were stored in ethanol (100%) for morphological and molecular genetic investigation.

### Sample locations

1. Hokkaido, Muroran, dark green algae growing on large rock in upper eulittoral area; coordinates 42°18ʹ25ʹ’N 140°58ʹ32ʹ’E; 13 September 2018.
2. Hokkaido, Yoichi, grey lichen growing extensively in spray zone on large quay wall; coordinates 43°14ʹ52ʹ’N 140°42ʹ35ʹ’E; 14 September 2018.

### Molecular genetic analyses

For inferring phylogenetic relationships and providing reference sequences, whole genomic DNA was extracted from 11 ethanol-fixed adult specimens of the family Ameronothridae using Chelex resin according to the adjusted protocols in Pfingstl et al. (2019). A ~ 566 bp long fragment of the mitochondrial DNA cytochrome oxidase subunit I (COI) was amplified as stated in Pfingstl et al. (2019) using the primer pair Mite COI-2F and Mite COI-2R (for primer composition see Otto and Wilson 2001). Furthermore, the complete 18S rRNA locus (~1733 bp) was amplified including primers shown in Dabert et al. (2010) along with the protocols in Skoracka and Dabert (2010). Subsequent DNA purification steps included enzymatic ExoSAP-IT (Affymetrix) and Sephadex G-50 resin (GE Healthcare). Cycle sequencing, using BigDye Sequence Terminator v3.1 kit (Applied Biosystems), was conducted according to Schäffer et al. (2008). Automatic capillary sequencing and sequence visualization was operated on an ABI 3130xl (Applied Biosystems) device. All generated sequences are deposited in GenBank under accession numbers MK883430 – MK883440 (COI) and MK880170 – MK880177 (18S) (see Table 1). MUSCLE (Edgar 2004), integrated in the software MEGA 7.0 (Kumar et al. 2016), was used to align sequences. Additionally, already published 18S fragment sequences were downloaded from GenBank for subsequent phylogenetic analysis (for Accession numbers see Table 1). To assess the best fitting substitution model, the implemented model search module in MEGA was applied. Model selection was based on lowest Bayesian Information Criterion (BIC) scores. Phylogenetic Bayesian inference (BI) and maximum likelihood (ML) analysis were conducted for the 18S locus in MrBayes 3.2 (Ronquist et al. 2012) and RaxML HPC v.8.0. (Stamatakis 2014), respectively. Posterior probabilities were generated from Metropolis-coupled Markov chain Monte Carlo simulations over 10 million generations in two independent runs, 7–8 chains, Kimura-2 parameter model + gamma (as a result of model test – see above) and 25% burn-in. To ensure stationary of all parameter and check for convergence, results were analysed in Tracer v.1.6. (Rambaut and Drummond 2007) and a 50% majority rule consensus tree was summarized from post burn in trees. For ML analysis, GTR+gamma model was employed on 10.000 bootstrap replicates to assess node support. ML and BI topologies were visualized in FigTree 1.4.2 (available at http://tree.bio.ed.ac.uk/software/figtree). Both approaches revealed the same overall topologies yielding in high node supports. However, only the BI tree is shown in this study. We only used the 18S locus for species delimitation and phylogenetic inference because relevant COI or 28S sequences were not available in GenBank.

**Table 1.** GenBank accession numbers for COI and 18S rRNA sequences used in this study.

	ID	COI	18S	Reference
***Ameronothrus yoichi* sp. n.**	JP14_A_01	MK883430		This study
	JP14_A_02	MK883431		
	JP14_A_03	MK883432		
	JP14_A_04	MK883433	MK880170	
	JP14_A_05	MK883434	MK880171	
	JP14_A_06	MK883435	MK880172	
	JP14_A_07	MK883436	MK880173	
	JP14_A_08	MK883437	MK880174	
	JP14_A_09	MK883438	MK880175	
	JP14_A_10	MK883439	MK880176	
	JP14_A_11	MK883440	MK880177	
*Fortuynia smiti*			MH285694	Pfingstl et al. (2019)
*Indopacifica pantai* ^a^			MH285692	
*Litoribates bonairensis*			MF997502	Pfingstl et al. (2017)
*Thasecazetes falcidactylus*			MF997501	
*Scapheremaeus nakanoshimensis*			LC367334	Bayartogtokh et al. (2018)
*Limnozetes rugosus*			KX397636	Krause et al. (2016)
*Hydrozetes lacustris*			KX397631	
*Paraquanothrus grahami* ^b^			KX397627	
*Schusteria littorea*			HM070345	Pepato et al. (2010)
*Scutovertex sculptus*			GQ864305	Dabert et al. (2010)
*Scapheremaeus palustris*			EU433989	Schaefer et al. (2010)
*Cymbaeremaeus cymba*			EU432201	Maraun et al. (2009)
*Thalassozetes shimojanai*			AB818524	Iseki and Karasawa (2014)

GenBank synonyms: ^a^ Selenoribatidae gen. sp. and ^b^*Aquanothrus* sp.

### Drawings and photographs

Preserved animals were embedded in Berlese mountant for microscopic investigation in transmitted light. Drawings were made with an Olympus BH-2 Microscope equipped with a drawing attachment. These drawings were first scanned, then processed and digitized with the free and open-source vector graphics editor Inkscape (https://inkscape.org).

The map of Hokkaido Island was made by GMT5.2.1 (http://gmt.soest.hawaii.edu/) and further modified with Adobe Photoshop 7.0.

For photographic documentation, specimens were air-dried and photographed with a Keyence VHX-5000 digital microscope.

Morphological terminology used in this paper follows that of Grandjean (1953), Schulte (1975) and Norton and Behan-Pelletier (2009).

## Results

### Descriptions of new taxa

**Family Ameronothridae** Vitzthum 1942**Genus *Ameronothrus*** Berlese 1896

Type species – *Eremaeus lineatus* Thorell, 1871

***Ameronothrus yoichi* sp. n**. Pfingstl and Shimano

Zoobank ID. urn:lsid:zoobank.org:pub:5B772E2C-7D5E-4C86-9955-AB84A84C50DA

#### 

##### Type material/locality

Holotype: adult female, Japan, Hokkaido, Yoichi, grey lichen in spray zone on quay wall; 13 September 2018, coll. S. Shimano, T. Pfingstl, S. Hiruta. (Number: TP-20,180,913–01). Two paratypes: adult male and adult female; same data as for holotype (numbers: TP-20,180,913–02, TP-20,180,913–03). All on microscopic slides, deposited at the Collection of Arachnida, Department of Zoology, National Museum of Nature and Science, Tokyo (NMST). Two additional paratypes from the same sample deposited in the collection of the Senckenberg Museum für Naturkunde Görlitz (SMNG) (number: 59,954).

##### Etymology

The specific name ‘*yoichi*” is given as noun in apposition and refers to its type locality.

##### Differential diagnosis

Colour dark brown, nearly black. Body length 525–600 µm. Notogastral cuticle with densely packed large nodules. Prodorsal lamellar keels converging, anteriorly fused by a semicircular translamella. Short clavate sensilli present. Interlamellar and exobothridial setae absent. Labiogenal articulation complete. All adanal setae located posteriorly of anal orifice. Primilateral setae on tarsus I absent. Dorsal companion seta *d* on genu I, II and III and all tibiae reduced to alveolus. Tarsal distal setae ending with a small nodule.

### Description

#### 

##### Measurements

Females (N = 2), length: 563–600 μm (mean 582 μm), width: 381–388 μm (mean 385 μm); males (N = 3), length: 525–556 μm (mean 535 μm), width: 306–356 μm (mean 325 μm).

##### Integument

Cuticle thin, easily deformable, showing large nodules. Cerotegument dense covering conspicuous nodular integument, consequently showing the same pusticulate pattern. Colour dark brown.

##### Prodorsum

**Figure 1.**
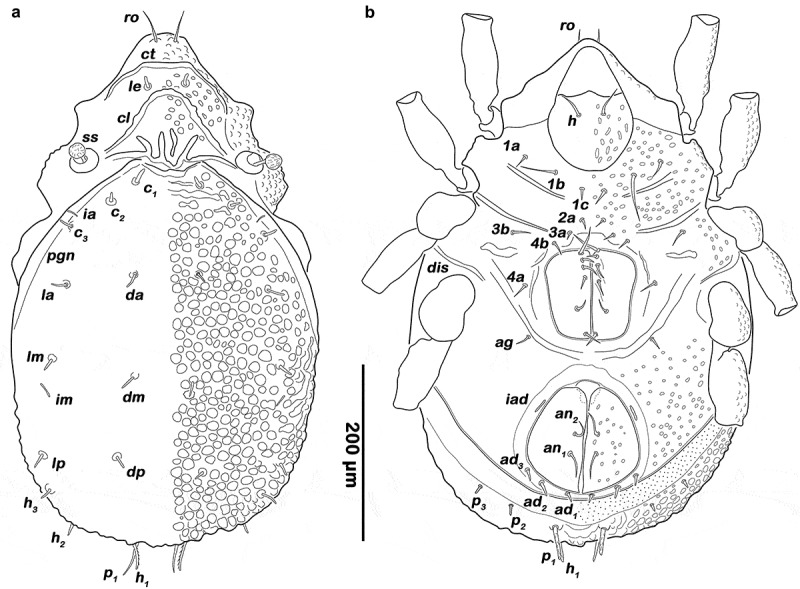
*Ameronothrus yoichi* sp. n. adult. (a) male dorsal view, legs omitted; (b) female ventral view, legs distal segments omitted. Scale bar valid for both depictions.

(Figure 1A, 2A) Rostrum rounded in dorsal view, demarcated from the remainder of prodorsum by strong transverse caudally arched ridge *ct*. Obvious lamellar keels (*cl*) converging, anteriorly fused by semicircular translamella. Transversal ridges originating from bothridia, forming medially several irregular longitudinal ridges next to the anterior border of notogaster. Rostral seta (*ro*) spiniform, long and smooth (approx. 35 µm). Lamellar seta (*le*) short, thickened, blunt and smooth (approx. 11 µm). Interlamellar seta (*in*) and exobothridial seta (*ex*) absent. Bothridium cup-like, orifice wide and circular. Sensillum (*ss*) short (approx. 30 µm), strongly clavate, globular head with inconspicuous linear elevations.

**Figure 2.**
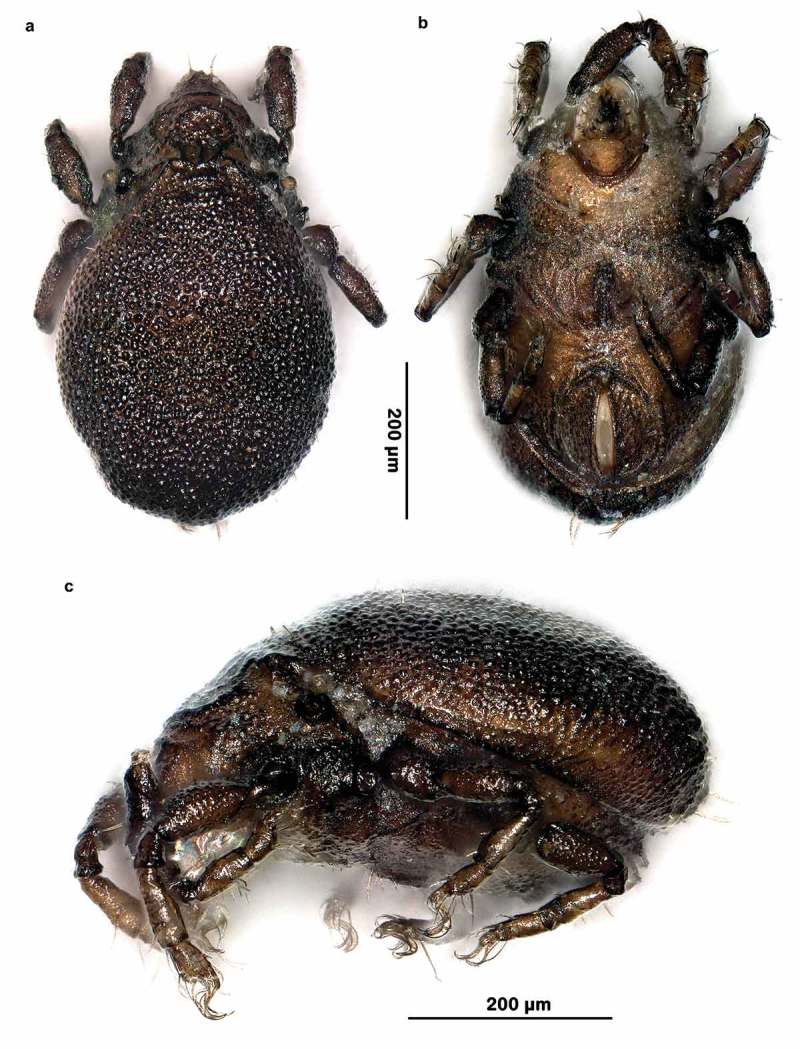
*Ameronothrus yoichi* sp. n. stereomicroscopic photographs of adult female. (a) dorsal view; (b) ventral view; (c) lateral view.

##### Gnathosoma

**Figure 3.**
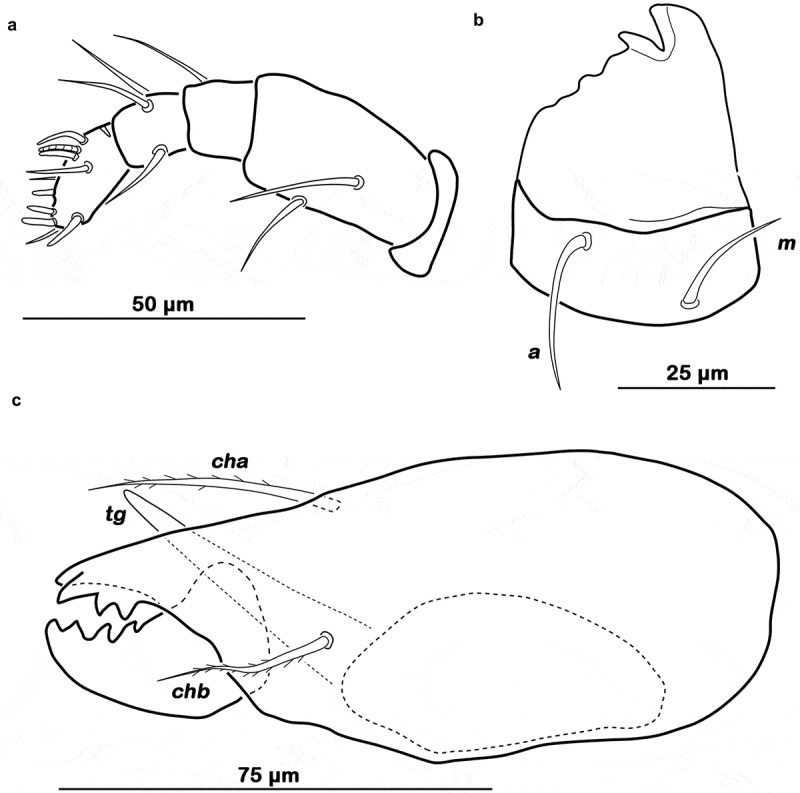
*Ameronothrus yoichi* sp. n. mouthparts adult. (a) left pedipalp antiaxial view; (b) left rutellum ventral view; (c) left chelicera antiaxial view.

Palp pentamerous 0–2–1–3-9 (solenidion not included), trochanter very short, femur by far longest segment, genu, tibia and tarsus of almost equal length (Figure 3A). Solenidion *ω* on palptarsus associated with eupathidium *acm*. Atelebasic rutellum. Distal part with wide paraxial tooth followed by a smaller tooth merging into a series of inconspicuous projections forming an undulating membranous edge (Figure 3B). Incision between rutellar teeth with darker sclerotization. Setae *a* (approx. 15 µm) and *m* (approx. 25 µm) long, robust and smooth. Mentum regular, seta *h* setiform, robust (approx. 23 µm). Labiogenal articulation complete. Chelicera chelate, mobile digit darker sclerotized; distinct strong interlocking teeth. Träghårds organ (*tg*) slender blunt lamella, slightly upward orientated. Seta *cha* and *chb* robust and barbed, *cha* longer (approx. 40 µm), *chb* shorter (approx. 23 µm) with a strong curvature (Figure 3C). No porose areas were detectable on any of the above-mentioned parts.

##### Gastronotic region

(Figure 1A, 2A) Oval in dorsal view, convex in lateral view; no distinct border between anterior median notogastral and prodorsal region. Ascleritic incision *pgn* visible along the lateral humeral area. Fifteen pairs of short, thickened and blunt notogastral setae (12–15 µm), *c_1-3_, da, dm, dp, la, lm, lp, h_1-3_, p_1-3_*; setae *p_1_* and *h_1_* conspicuously longer than others, the latter unilaterally barbed. Five pairs of notogastral lyrifissures present but difficult to trace due to the rough cuticular surface; *ia* between seta *c_2_* and *c_3_*, but closer to the latter; *im* between seta *lm* and *lp; ih* laterad and anterior to *h_3_*; lyrifissures *ip* and *ips* laterally of seta *p_3_* and *p_2_* respectively. Orifice of the opisthonotal gland (*gla*) not traceable due to heavy ornamention.

##### Lateral aspect

(Figure 2C) Pedotectum I and II absent. Discidium *dis* between acetabulum III and IV, developed as conspicuous rounded ridge.

##### Podosoma and venter

(Figure 1B, 2B) Epimeral setation 3–1–2–2, all setae setiform and smooth, seta *1b* conspicuously longer (approx. 37 µm) than others (10–21 µm). Genital orifice large, rectangular with rounded corners, anteriorly broader. Six pairs of genital setae arranged in longitudinal rows, whereas the fifth pair slightly laterally displaced and first slightly longer than others (30–37 µm). One pair of short, setiform aggenital setae *ag*. Strongly curved obvious transversal ridge between genital and anal opening. Anal valves were triangular but strongly rounded. Outer part of preanal organ rectangular with rounded edges, inner part shaped like a transverse bar. Two pairs of thin anal setae, *an_1-2_* (19–25 µm), inserting close to the median border. Three pairs of short adanal setae, *ad_1-3_* (approx. 12 µm), all located posteriorly of anal orifice. Lyrifissure *iad* flanking anterior third of anal plates.

##### Legs

**Figure 4.**
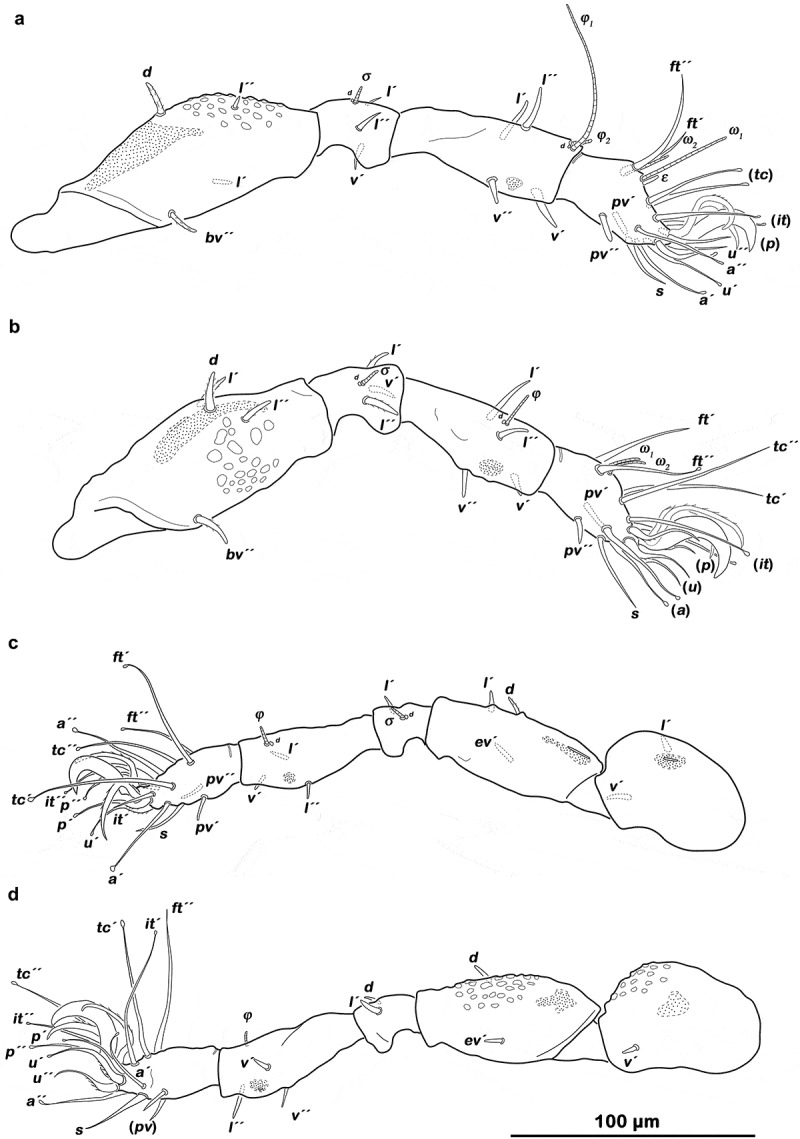
*Ameronothrus yoichi* sp. n. adult legs. (a) right leg I antiaxial view; (b) right leg II antiaxial view; (c) left leg III dorsolateral view of antiaxial aspect; (d) left leg IV ventrolateral view of antiaxial aspect.

(Figure 4) Ambulacrum tridactylous, median claw broad and strong, lateral claws weaker developed and dorsally slightly dentate. Extensive brachytracheae with slit-like stigmata on the dorsal paraxial face of all femora and tracheal sacculi ventrally on all tibiae and dorsally on trochanter III and IV. Dorsal companion seta *d* (usually associated with solenidia) on genu I, II and III and all tibiae reduced to alveolus. Primilateral setae of tarsus I absent. Tectal (*tc*) and iteral (*it*) setae as well as most other terminal tarsal setae with spoon-shaped or nodular tips (these are sometimes difficult to observe). Famulus *ε* on tarsus I rod-like, blunt and next to solenidion *ω_1_*, solenidion *ω_2_* shorter and in slight paraxial position. Solenidia *ω_1_* and *ω*_2_ on tarsus II adjacent. Chaetome and solenidia see Table 2.

**Table 2.** *Ameronothrus yoichi* sp. n. development of leg setation and solenidia from larva to adult (except for tritonymph).

	Instars	Trochanter	Femur	Genu	Tibia	Tarsus	Chaetome	Solenidia
Leg I	larva	-	*d, bv´´*	(*l), d, σ*	(*l), v´, d, φ_t_*	(*pl), s*, (*a*), (*u*), (*p*), (*tc*), (*ft), ε, ω_t_*	0–2–3–4-14	1–1–1
	protonymph	-	-	-	-	*ω_2_*	0–2–3–4-14	1–1–2
	deutonymph	-	*l´´*	-	*φ _2_*	-	0–3–3–4-14	1–2–2
	adult	-	*l´*	*v´, d* lost	*v´´, d* lost	(*it*)	0–4-3–4-16	1–2–2
Leg II	larva	-	*d, bv´´*	(*l*), *d, σ*	*l´, v´, d, φ*	(*pv), s*, (*a*), (*u*), (*p*), (*tc*), (*ft), ω*	0–2-3–3-13	1–1–1
	protonymph	-	-	-	-	-	0–2-3-3-13	1–1–1
	deutonymph	-	*l´´*	-	*l´´*	-	0–3-3–4-13	1–1–2
	adult	-	*l´*	v´, *d* lost	*v´´, d* lost	(*it*)	0–4-3–4-15	1–1-2
Leg III	larva	-	*d, ev´*	*l´, d, σ*	*v´´, d, φ*	(*pv), s*, (*a*), (*u*), (*p*), (*tc*), (*ft*)	0–2–2–2-13	1–1-0
	protonymph	*v´*	-	-	-	-	1-2–2-2-13	1-1-0
	deutonymph	*l´*	-	-	-	-	2-2-2-2-13	1-1-0
	adult	-	*l´*	*d* lost	(*l), d* lost	(*it*)	2-3-1–3-15	1-1-0
Leg IV	protonymph	-	-	-	-	(*pv*), (*u*), (*p), ft´´*	0–0–0–0-7	0-0-0
	deutonymph	-	*d, ev´*	*d, l´*	*v´, d, φ*	*s*, (*a*), (*tc*)	0–2-2-2-12	0–1-0
	adult	*v´*	-	-	(*l), d* lost	(*it*)	1-2-2-3-14	0–1-0

First development of setae characterized by letters, () = pairs of setae, – = no change with regard to preceding stage, *d* lost = dorsal companion seta reduced.

### Common features of juvenile stages

#### 

##### 

Apheredermous. Colour dark brown. Integument soft and strongly plicate (“nymphes plissées” in the sense of Grandjean 1953). Prodorsum triangular, rostrum rounded, cerotegument overall finely granular. Rostral seta (*ro*) thin, long and smooth, lamellar (*le*) and interlamellar setae (*in*) thickened, blunt and barbed. Exobothridial seta (*ex*) small and smooth. Bothridia small cup-like. Sensillum short, clavate, head with inconspicuous linear elevations. Hysterosoma oval in dorsal view, slightly convex in lateral view. Notogastral setae thickened and barbed, posterior setae longest, median dorsal setae shortest. Orifice of opisthonotal gland *gla* located in latero-ventral folds on a level with anterior third of anal valves. Legs monodactylous, with slightly invaginated porose areas on the same leg segments and position as brachytracheae in adults. Solenidia on genu and tibia always associated with dorsal companion setae (both approx. the same size).

##### Larva

(N = 1): length 249 µm.

Prodorsal region. Sensillum short clavate, head drop-shaped with a pointed tip.

Gastronotic region. Twelve pairs of notogastral setae; *c_1-3_, da, dm, dp, la, lm, lp, h_1-3_*.

Ventral region of idiosoma. Epimeral setation 2(3)-1-2, third seta on epimeron 1(given in parentheses) developed as protective seta (number in parentheses includes this seta) covering Claparède’s organ. No anogenital setae present in this stage. Posterior third of anal orifice framed by smooth and short seta *h_3_* and smooth and long seta *h_2_*.

Legs. Chaetome and solenidia see Table 2.

##### Protonymph

(N = 1): length 286 µm.

Prodorsal region. Sensillum short clavate, head globular without pointed tips from this stage.

**Figure 5.**
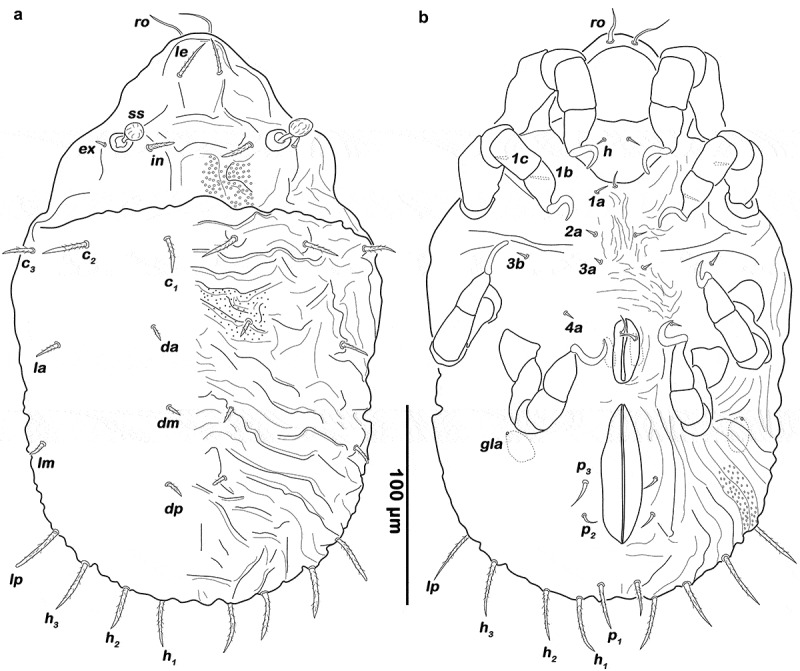
*Ameronothrus yoichi* sp. n. protonymph. (a) dorsal view; (b) ventral view. Scale bar valid for both depictions.

**Figure 6.**
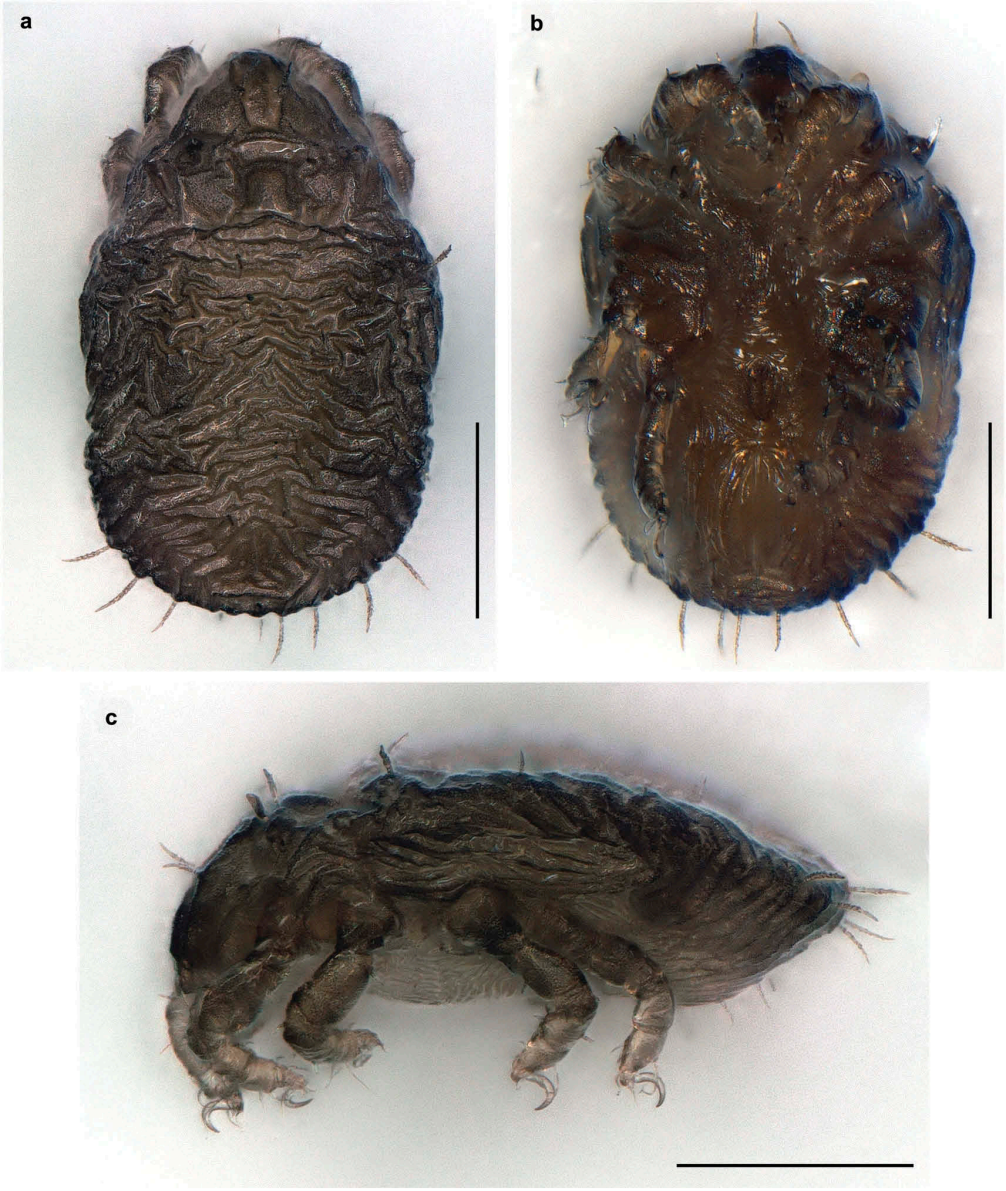
*Ameronothrus yoichi* sp. n. stereomicroscopic photographs of protonymph. (a) dorsal view; (b) ventral view; (c) lateral view. Scale bars 100 µm.

Gastronotic region (Figure 5A, 6A, 6C). Fifteen pairs of notogastral setae; *c_1-3_, da, dm, dp, la, lm, lp, h_1-3_, p_1-3_*.

Ventral region of idiosoma (Figure 5B, 6B). Epimeral setation 3–1-2-1. One pair of short genital setae, placed on the anterior half of genital valves. Adanal and anal setae not developed. Setae *p_3_* and *p_2_* short and smooth framing posterior half of anal orifice, seta *p_1_* longer and slightly barbed.

Legs. Chaetome and solenidia see Table 2.

##### Deutonymph

(N = 5): length 356–425 µm (mean 388 µm).

**Figure 7.**
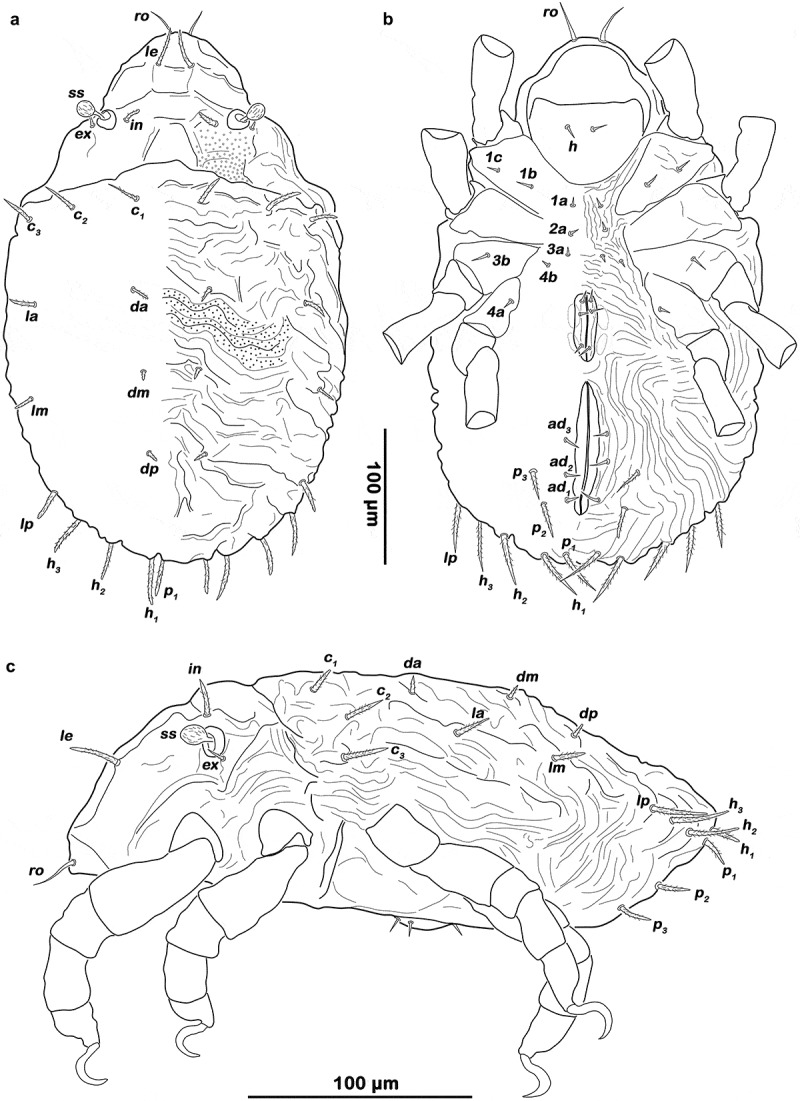
*Ameronothrus yoichi* sp. n. deutonymph. (a) dorsal view; (b) ventral view, distal leg segments omitted; (c) lateral view, legs drawn simplified.

Gastronotic region (Figure 7A, 7C). Fifteen pairs of notogastral setae, same positions and shapes as in protonymph.

Ventral region of idiosoma (Figure 7B). Epimeral setation 3–1-2–2, seta *4b* added in a median position. Two pairs of short genital setae aligned in a longitudinal row. Three pairs of short, smooth adanal setae *ad_1-3_* flanking anal orifice.

Legs. Chaetome and solenidia see Table 2.

***Tritonymph*** no data available

### Ecological data

**Figure 8.**
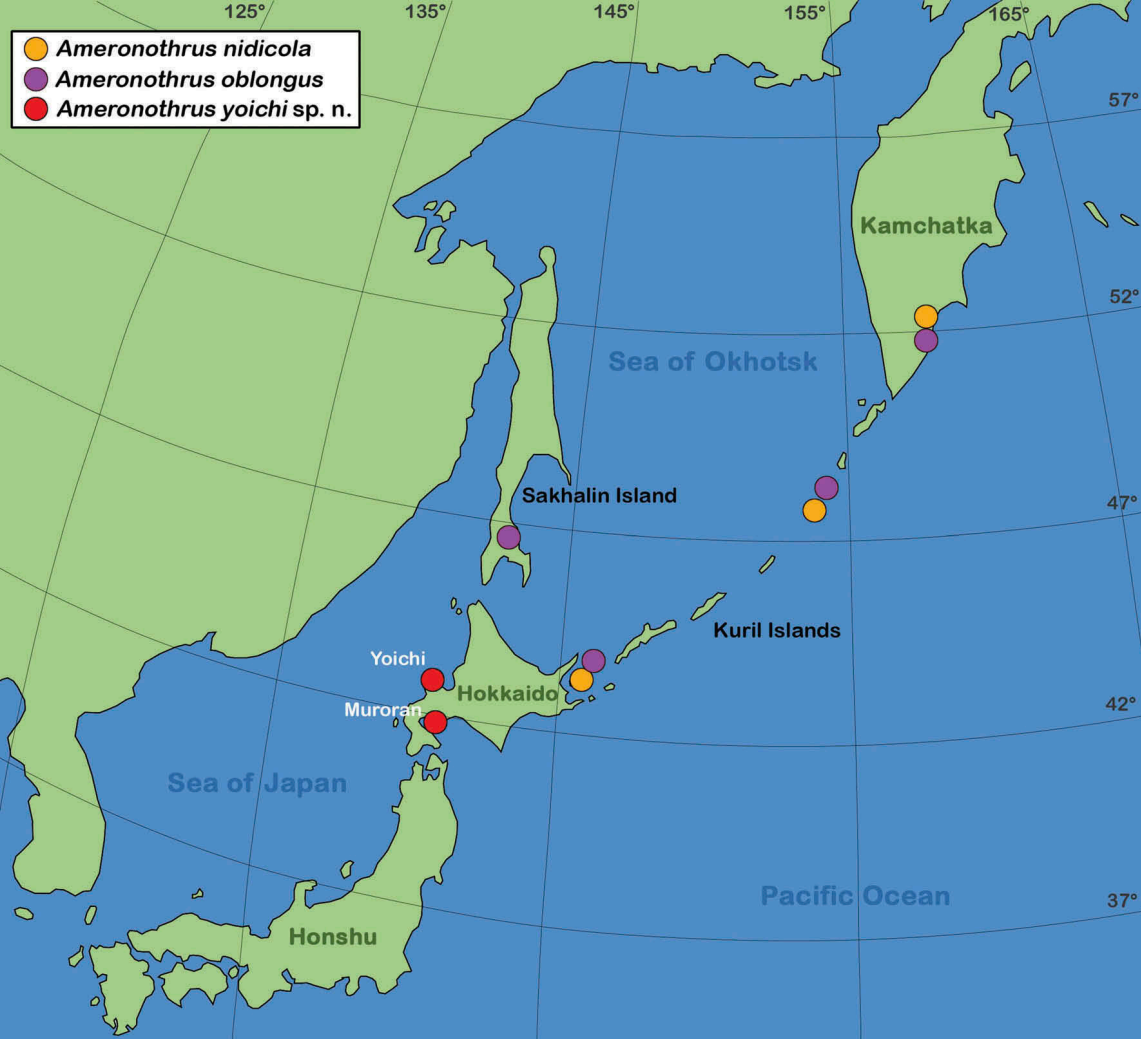
Distribution of *Ameronothrus* species in the northern Asian Pacific region.

The specimen from Muroran (Figure 8) was found in black algae growing on a large rock in the upper eulittoral zone of a rough coastal area with strong wave action. The sea at this location belongs to the North Pacific Ocean but still is influenced by the warm Tsugaru current which flows eastwards through the strait of Tsugaru (between Hokkaido and Honshu connecting the Sea of Japan with the Pacific Ocean). The air temperature at Muroran ranges from −2.6°C in February to 20.1°C in August, while the sea surface temperature ranges from approximately 2°C in the coldest month to 23°C in the warmest month (Table 3).

**Table 3.** Climate data for the two record locations on Hokkaido. SST = sea surface temperature range derived from historical data (www.seatemperature.org); as there were no direct data for Muroran, available data for the next closest town, named Date (20 km away), was taken as reference. AAT = average air temperature for the year 2018 taken from Japan Meteorological Agency (www.jma.go.jp).

	Muroran/Date			Yoichi		
	SST		AAT	SST		AAT
	Min°C	Max°C	°C	Min°C	Max°C	°C
Jan	2,5	9	−1,2	4,9	9,3	−3,4
Feb	2,1	5,2	−2,6	4,9	7,2	−5
Mar	1,8	5,1	3	4,8	7	1,7
Apr	3,6	7,7	7,2	5,9	9,6	7,3
May	4,8	12,5	11,4	7,5	13,3	11,8
Jun	10	19,3	14,7	11,7	18,3	15,9
Jul	15,3	22	18,9	16,5	21,3	20,3
Aug	19,6	23,2	20,1	20,4	23,3	20,2
Sep	17,5	22,9	18,5	18,4	23,4	16,9
Oct	13,8	18,7	13,9	14,2	19,4	11,5
Nov	9,7	14,8	7,9	9,7	15,1	5,3
Dec	5,1	12,2	0,6	6,8	11,4	−1,9

The population from Yoichi was collected from large layers of grey lichen growing on a massive quay wall. The sample was taken in the supralittoral area, which is only exposed to spray from the seawater. This location lies at the Sea of Japan (Figure 8) in an area that is influenced by the warm northwards flowing Tsushima current. The air temperature in Yoichi ranges from −5°C in the winter to 20°C in the summer, whereas sea surface temperature is the lowest in March with approx. 4.8°C and the highest in September with 23.4°C (Table 3).

## Genetic data

**Figure 9.**
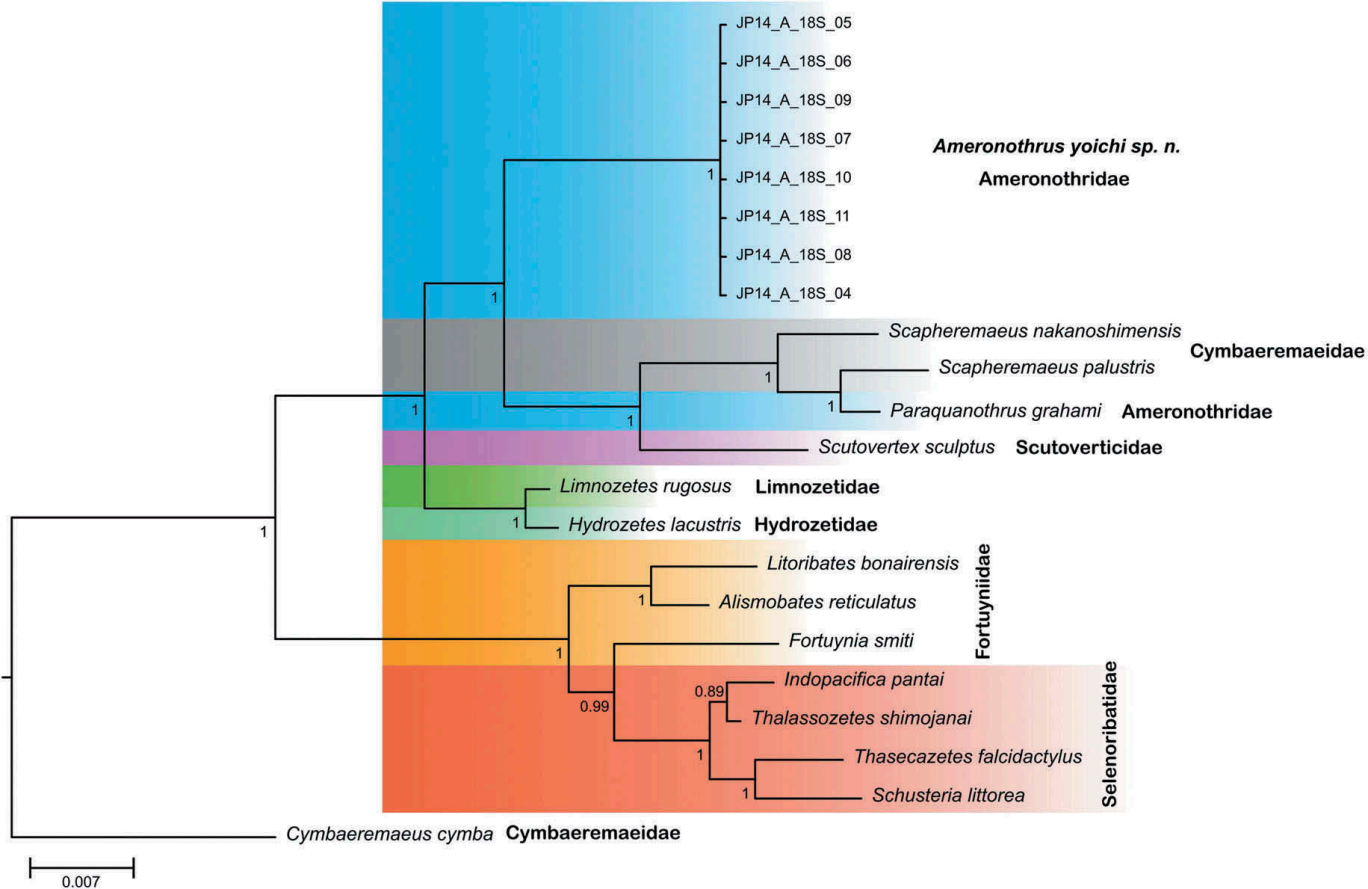
Bayesian inference tree based on 18S rRNA sequences (1751 bp). Different families are highlighted in colour. Individual sequences of the newly described *Ameronothrus yoichi* sp. n. species are labelled from JP14_A_03 – JP14_A_11. References and accession numbers for newly created and downloaded sequences can be found in Table 1.

COI as well as 18S rRNA sequence data confirm all investigated specimens as members of *A. yoichi*. The Bayesian inference tree based on 18S rRNA further shows a well-separated *A. yoichi* clade but places it in paraphyly to *Paraquanothrus grahami* Norton & Franklin, 2018, a supposed member of the Ameronothridae (Figure 9). Additionally, *A. yoichi* as well as *P. grahami* are placed outside other members of the superfamily Ameronothroidea, namely the Selenoribatidae and Fortuyniidae that are represented as a monophyletic group in the tree.

## Discussion

### Systematics

The new species *Ameronothrus yoichi* can be easily distinguished from its congeners by its unique prodorsal ridge structure, the conspicuously pusticulate body surface showing densely packed large nodules and the loss of dorsal companion setae *d* on all genua in the adult stage. Most other characteristics do overlap with certain species and hence can only be used in specific combinations for determination. Schulte (1975) classified four groups of *Ameronothrus* species based on morphological correlations, (I) the *Ameronothrus marinus* group containing *A. marinus* (Banks, 1896), *A. bilineatus* (Michael, 1888), *A. schusteri* Schubart, 1970 and *A. schubarti* Weigmann & Schulte 1975, (II) the *Ameronothrus maculatus* group consisting of *A. maculatus* (Michael, 1882) and *A. schneideri* (Oudemans, 1903), (III) the *Ameronothrus lineatus* group with *A. lineatus* (Thorell, 1871) and *A. nigrofemoratus* (L. Koch, 1879) and (IV) the monotypic *Ameronothrus lapponicus* Dalenius, 1963 group. *Ameronothrus yoichi* does not share any group-specific characters with the *A. marinus* and the *A. lapponicus* group. It shows the same loss of dorsal tibial setae *d* as the *A. lineatus* group (whereas in *A. yoichi* the alveoli of these setae are still present), but differs in all the other group traits. It shares the lack of primilateral tarsal setae (*pl*), the reduction of the ascleritic incision *pgn* and the complete labiogenal articulation with the *A. maculatus* group, but shows a differing anal setation with two instead of only one pair of setae. Based on these conformities, *A. yoichi* seems to be closest related to *A. maculatus* and *A. schneideri*, though there is no complete match with the group. Unfortunately, the descriptions of *A. dubinini, A. nidicola* and *A. oblongus* (Sitnikova 1975, 1977) are not detailed enough for a thorough comparison and classification into one evolutionary group in the sense of Schulte (1975); therefore, a clear assessment of phylogenetic relationships within *Ameronothrus* is not feasible yet.

The same applies to juvenile morphology as information on the above-mentioned three species and *A. nigrofemoratus* and *A. schubarti* is lacking. Nevertheless, a comparison with known *Ameronothrus* juveniles shows that *A. yoichi* exhibits the same developmental patterns and formulas, e.g. prodorsal, notogastral, epimeral and anogenital setation, but differs from other species in certain characteristics. *Ameronothrus marinus, A. bilineatus* and *A. schneideri* already lack a trichobothrium in the juvenile stages and *A. lineatus* and *A. lapponicus* show uniform shapes of notogastral setae (e.g. Schulte 1975; Ermilov et al. 2012) whereas *A. yoichi* juveniles show enlarged and broadened, posterior notogastral setae. A similar condition can only be found in the nymphs of *A. maculatus*, but here seta *lp* is not thickened and enlarged as it is in *A. yoichi*.

In a larger phylogenetic context, the present data places *A. yoichi* in a paraphyletic position to *Paraquanothrus grahami*, the second supposedly member of Ameronothridae. This contrasts with the recently suggested close relationship of Aquanothrinae and Ameronothridae (Norton and Franklin 2018) and questions its inclusion in the latter. The suggested independent evolutionary origin of the littoral Ameronothridae, Fortuyniidae and Selenoribatidae (Krause et al. 2016; Pfingstl 2017), all members of the superfamily Ameronothroidea, on the other hand, is supported as shown by their paraphyletic placement. The monophyletic origin of Fortuyniidae and Selenoribatidae was already confirmed by certain studies (Iseki and Karasawa 2014; Krause et al. 2016) and is further affirmed by the present data. Nonetheless, comprehensive molecular genetic studies, including many more taxa, are necessary to solve found discrepancies and to verify indicated phylogenetic positions.

### Ecology

Schulte et al. (1975) postulated four ecological groups of ameronothrid mites: (I) stenotopic inhabitants of the marine littoral populating characteristic salinity ranges, (II) eurytopic inhabitants of the littoral living in saline and brackish waters, (III) holeurytopic inhabitants of the littoral living in limnic, brackish, marine and terrestrial habitats and (IV) stenotopic inhabitants of inland regions dwelling exclusively in terrestrial habitats. Based on the few present records of *A. yoichi* it is difficult to assess the ecology of this species but the populations were found in the upper marine eulittoral and supralittoral area hence a strictly terrestrial lifestyle (group IV) can be excluded. Additionally, they were found at relatively low latitudes and species occurring at lower latitudes usually tend to be stenotopic and restricted to marine environments (Schulte et al. 1975; Marshall and Convey 2004); therefore, *A. yoichi* most likely belongs to group I or II.

Several *Ameronothrus* species, i.e. *A. marinus, A. schusteri, A. lineatus, A. maculatus* and *A. schubarti* are known to live mainly in the sediment-free rock littoral (Schulte et al. 1975) and the same may apply to *A. yoichi* as both investigated populations were found restricted to this habitat. *Ameronothrus yoichi* shows three claws on each leg, which further points to the rocky substrate as the main habitat because tridactylous *Ameronothrus* species are usually found on solid substrates while monodactylous species are mainly restricted to soft substrates (Schulte 1975).

All *Ameronothrus* species are microphytophagous and can be classified into three groups, namely, lichenivorous species feeding on lichen growing on hard substrates of the coast or inland, algivorous species subsisting on diverse algae from hard coastal substrates and fungivorous species feeding on fungal growth in the upper soil layer of salt marshes (Schulte 1976). Based on its predominant occurrence in lichen on a hard substrate, *A. yoichi* most likely belongs to the lichenivorous group. However, feeding experiments in the lab would be necessary to confirm such a food preference.

In all known cases, *Ameronothrus* species are larviparous (Bücking et al. 1998) or at least ovoviviparous (Søvik 2003), which means that larvae hatch immediately after deposition. The same is true for *A. yoichi* as we found fully developed larvae contained within eggshells inside the body of a female.

### Biogeographic aspects

In a global context, the occurrence of *A. yoichi* at a latitude of approximately 42 degrees North is nothing unusual because distributions of most *Ameronothrus* species stretch further south (Schulte 1975). In the Asian Pacific region, on the other hand, represents the record of *A. yoichi* on Hokkaido the southernmost occurrence of an *Ameronothrus* species. The congeneric *A. nidicola* and *A. oblongus* were reported from Kunashir Island (Klimov 1998), which is very close to Hokkaido but lies approximately two degrees more northern (see Figure 8). The distribution of both species stretches further north with records from Sakhalin Island, Lovushki and the Peninsula of Kamchatka (Ryabinin 2015). This pattern indicates that *A. nidicola* and *A. oblongus* are adapted to colder climates than *A. yoichi* whereas it is not clear yet how far the distribution of the latter reaches into the South. Records from Honshu are lacking, but occurrences in the north and hence colder regions of this Japanese Island should be considered. Water temperatures seem to play an important role in shaping the distributions of *Ameronothrus* as shown in *A. lineatus* and *A. nigrofemoratus*. These species reach the latitude of 38° North at the Pacific American shore where consistently cold waters prevail while they only reach a latitude of 50° North at European coasts where comparable water temperatures are present (Schulte 1975). Japanese coasts are strongly influenced by the warm Kuroshio Current and its branch the Tsushima Current and these warm waters may limit the southern distribution of Asian Pacific *Ameronothrus* species.

However, biogeographic studies combined with local climate data would be necessary to prove such a correlation. Unfortunately, most studies on *Ameronothrus* species provide the locations of the species found, but no detailed information on the climate prevailing at these locations, therefore only vague conclusions might be drawn. In the light of global warming, local climates may change rapidly and hence distributions of *Ameronothrus* species may alter, therefore providing reference data is the first step in predicting these shifts.
